# A Five-Species Jungle Game

**DOI:** 10.1371/journal.pone.0157938

**Published:** 2016-06-22

**Authors:** Yibin Kang, Qiuhui Pan, Xueting Wang, Mingfeng He

**Affiliations:** 1 School of Mathematical Science, Dalian University of Technology, Dalian, 116024, China; 2 School of Innovation Experiment, Dalian University of Technology, Dalian, 116024, China; Shanxi University, CHINA

## Abstract

In this paper, we investigate the five-species Jungle game in the framework of evolutionary game theory. We address the coexistence and biodiversity of the system using mean-field theory and Monte Carlo simulations. Then, we find that the inhibition from the bottom-level species to the top-level species can be critical factors that affect biodiversity, no matter how it is distributed, whether homogeneously well mixed or structured. We also find that predators’ different preferences for food affect species’ coexistence.

## Introduction

Cyclically dominant systems play a prominent role in nature, especially in explaining biological diversity[1–5]. As the simplest cyclical interaction system that contains three species, the rock-paper-scissors game can explain typical population oscillatory behavior and other indications, i.e., marine benthic systems[6], plant communities[7–11] and microbial populations[2,12–15]. The rock-paper-scissors game is also reflected in the strategy choice on biological methods, for example, the mating strategy of side-blotched lizards[16], the regular oscillations of the numbers of collared lemmings[17] and pacific salmon[18]. In other words, biological diversity can be interpreted by the invasion between species [19, 20]. Extensions of the classical rock-paper-scissors game to more than three strategies have been popular issues in recent research. Avelino *et al*. investigated the three-dimensional predator-prey model with four or five species, showing the spatial distribution of *Z*_*N*_ Lotka-Volterra competition models using stochastic and mean field theory simulations[21]. Dobrinevski *et al*. considered an asymmetric ecological model with four strategies, which contains a three-strategy cycle and a neutral alliance of two strategies, showing that the model exhibits a mobility-dependent selection of either the three-strategy cycle or the neutral pair[22]. Durney *et al*. discussed the evolution of characters of a cyclically competing predator-prey system with four or more species[23]. Feng *et al*. observed self-organization spiral waves of a cyclic five-species system using direct simulations and nonlinear partial differential equations[24]. Intoy *et al*. focused on the extinction processes in a cyclic four-species system[25]. In our previous work, we studied the evolution properties of a cyclic five-strategy system with two different invasion routes[26], and the group interactions of the system have been discussed[27–29]. Knebel *et al*. analyzed the coexistence and survival scenarios of Lotka-Volterra networks with both a cyclic four-species system and a cyclic five-species system[30]. Laird *et al*. provided numbers for possible competitive topologies for a cyclic five-species system, showing the different coexistences[31]. Li *et al*. analyzed the evolution properties of the N-species Jungle game, which is a special cyclic competing system by mean-field theory[32]. Spatial effects and time delay have influences on cyclically dominant systems, Sun et al. have made important contributions in these fields[33–40].

Cyclic dominance of species plays an important role in biology system in nature[2,16]. The higher-level species invade the lower-level species; however, the bottom-level species may invade the top-level species. For example, the lowest level can be thought of as bare space, which is invaded by grasses and other pioneering spaces, which are invaded in turn by small shrubs and finally forest trees. The forest is destroyed by fire to return to the bare space condition.

Cyclic dominance of strategies also plays an important role in sociology, e.g. four strategies ALLC, ALLD, TFT(Tit For Tat) and WSLS(Win Stay Lose Shift) in a repeated prisoner’s dilemma[41]. Strategy ALLD can invade strategies WSLS and ALLC, and be invaded by strategy TFT, just like the top-level species in our model. Strategy WSLS can invade strategies ALLC and TFT, and be invaded by strategy ALLD, just like the second-level species in our model. Strategy ALLC can invade strategy TFT, and be invaded by strategies ALLD and WSLS, just like the third-level species in our model. Strategy TFT can invade strategy ALLD, and be invaded by strategies WSLS and ALLD, just like the bottom-level species in our model. From this we know that strategies ALLD and WSLS can both invade two different strategies. However, as strategy WSLS can be invaded by strategy ALLD, strategy ALLD has the higher level than strategy WSLS. Similarly, strategy ALLC has the higher level than strategy TFT.

In our paper, we investigate the five-species Jungle game in the framework of evolutionary game theory. The Jungle game are based on a traditional Chinese board game. We investigate the coexistence of the species using mean-field theory and Monte Carlo simulation and discuss the biological significance of two special examples. In Ref. [32], the authors find that species may become extinct in seconds without considering the impact of the changing invasion rate on the system, which is not valid for ecological systems in nature. In our paper, we find that the coexistence of species is related to the invasion rate between species. Species are well mixed in mean-field theory; we give all of the appropriate functional relationships of invasion rates for every coexistence state of the system, respectively. In the Monte Carlo simulation, we discuss the impacts of different invasion rates on the system. We define the primary food of a chosen species as the next-level species. Other species, not the chosen species and its next-level species, are the sub-food of the chosen species. We find that predators’ different preferences for food affect the coexistence of species. The more the predation rate on predators' sub-prey approaches 1.149 times the rate on the primary prey, the lower is the area in the parameter space (*p*_2_, *s*) that makes all five species coexist.

## Model

**Fig 1:**
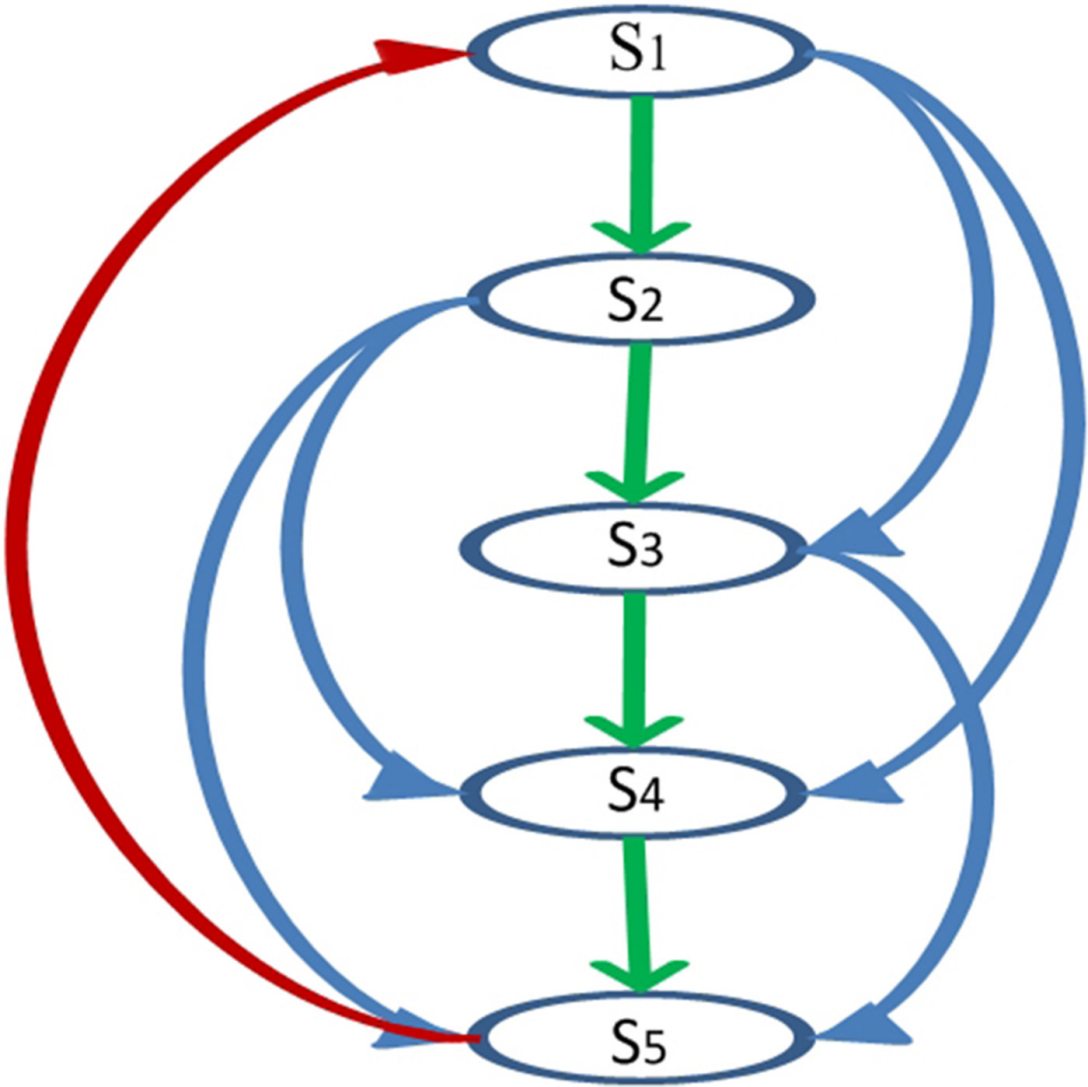
(color online). The relationships of five species in the Jungle game. Arrows point from predator to prey. *S*_1_ and *S*_2_ can prey three species and be hunt by one species; *S*_3_ can prey two species and be hunt by two species; *S*_4_ and *S*_5_ can prey one species and be hunt by three species.

The traditional Chinese board game, the Jungle game, ranks animals on the board as 1, 2, …. The animal ranking in our paper, from strongest to weakest, is *S*_1_-Elephant, *S*_2_-Tiger, *S*_3_-Wolf, *S*_4_-Cat and *S*_5_-Rat. The above jungle game between individuals of five species *S*_1_, *S*_2_, *S*_3_, *S*_4_ and *S*_5_ is established to describe cyclic competition and reproduction according to the following invasion rules (see Fig 1):

$$\begin{array}{l} S_{1}+S_{2}\overset{K_{1,2}}{\to }S_{1}+S_{1}\\ S_{1}+S_{3}\overset{K_{1,3}}{\to }S_{1}+S_{1}\\ S_{1}+S_{4}\overset{K_{1,4}}{\to }S_{1}+S_{1}\\ S_{2}+S_{3}\overset{K_{2,3}}{\to }S_{2}+S_{2}\\ S_{2}+S_{4}\overset{K_{2,4}}{\to }S_{2}+S_{2}\\ S_{2}+S_{5}\overset{K_{2,5}}{\to }S_{2}+S_{2}\\ S_{3}+S_{4}\overset{K_{3,4}}{\to }S_{3}+S_{3}\\ S_{3}+S_{5}\overset{K_{3,5}}{\to }S_{3}+S_{3}\\ S_{4}+S_{5}\overset{K_{4,5}}{\to }S_{4}+S_{4}\\ S_{1}+S_{5}\overset{K_{5,1}}{\to }S_{5}+S_{5} \end{array}$$

Species *S*_1_ invades *S*_2_, *S*_3_ and *S*_4_ with invasion rates *K*_1,2_, *K*_1,3_ and *K*_1,4_; Species *S*_2_ invades *S*_3_, *S*_4_ and *S*_5_ with invasion rates *K*_2,3_, *K*_2,4_ and *K*_2,5_; Species *S*_3_ invades *S*_4_ and *S*_5_ with invasion rates *K*_3,4_ and *K*_3,5_; Species *S*_4_ invades *S*_5_ with invasion rate *K*_4,5_; Species *S*_5_ invades *S*_1_ with invasion rate *K*_5,1_. In the traditional Chinese board game rules, the rat can capture the elephant, as it can crawl in the elephant’s ear and gnaw at its brain. Here, *K*_*i*, *j*_ means the rate that species *S*_*i*_ invades species *S*_*j*_.

## Mean-Field Theory

Considering what happens without the spatial constraints in effect, we study a well-mixed system using mean-field approximation. The dynamics of the system are described by the following equations, with the densities *x*_*i*_ of species *S*_*i*_:

$$\begin{array}{l} \partial_{t}x_{1}=x_{1}\left(K_{1,2}x_{2}+K_{1,3}x_{3}+K_{1,4}x_{4}-K_{5,1}x_{5}\right)\\ \partial_{t}x_{2}=x_{2}\left(-K_{1,2}x_{1}+K_{2,3}x_{3}+K_{2,4}x_{4}+K_{2,5}x_{5}\right)\\ \partial_{t}x_{3}=x_{3}\left(-K_{1,3}x_{1}-K_{2,3}x_{2}+K_{3,4}x_{4}+K_{3,5}x_{5}\right)\\ \partial_{t}x_{4}=x_{4}\left(-K_{1,4}x_{1}-K_{2,4}x_{2}-K_{3,4}x_{3}+K_{4,5}x_{5}\right)\\ \partial_{t}x_{5}=x_{5}\left(K_{5,1}x_{1}-K_{2,5}x_{2}-K_{3,5}x_{3}-K_{4,5}x_{4}\right) \end{array}$$ (1)

Let *A* = *K*_1,3_*K*_2,5_+*K*_2,3_*K*_5,1_−*K*_1,2_*K*_3,5_, *B* = *K*_1,4_*K*_2,5_+*K*_2,4_*K*_5,1_−*K*_1,2_*K*_4,5_ and *C* = *K*_1,4_*K*_3,5_+*K*_3,4_*K*_5,1_−*K*_1,3_*K*_4,5_, if *ABC* ≠ 0, we obtain the following conclusions (for proof, see Appendix):

1. When *A* > 0 and *B* > 0, three species, *S*_1_, *S*_2_ and *S*_5_, can stably coexist and the dynamic system is equivalent to a stable rock-paper-scissors system with three species.
2. When *A* < 0 and *C* > 0, three species, *S*_1_, *S*_3_ and *S*_5_, can stably coexist and the dynamic system is equivalent to a stable rock-paper-scissors system with three species.
3. When *B* < 0 and *C* < 0, three species, *S*_1_, *S*_4_ and *S*_5_, can stably coexist and the dynamic system is equivalent to a stable rock-paper-scissors system with three species.
4. When *AB* < 0 and *AC* > 0, all five species can stably coexist in this system.

In summary, three rock-paper-scissors topological structures, *S*_1_*S*_2_*S*_5_, *S*_1_*S*_3_*S*_5_ and *S*_1_*S*_4_*S*_5_, may emerge with different values of invasion rates. Moreover, all five species can stably coexist when the invasion rates meet the conditions *AB* < 0 and *AC* > 0.

## Example 1

In this five-species jungle game, 10 parameters are used to describe the invasion rates between species. Already in this low dimension it is difficult to draw intuitive conclusions. Thus, we simplify the model to explore the biological significance and it should be note that we do not consider the trophic loss in this model. In such a jungle game, species *S*_1_ is located on the top level of the food chain, and species *S*_5_ is located on the bottom of the food chain. Each species feeds on the species at lower levels. We define the primary food of a chosen species as the next-level species. Other species, not the chosen species and its next-level species, are the sub-food of the chosen species. The bottom-level species have an inhibitory effect on the top-level species; thus, the bottom-level species invade the top-level species. For example, the lowest level can be thought of as bare space, which is invaded by grasses and other pioneering spaces, which are invaded in turn by small shrubs and finally forest trees. The forest is destroyed by fire to return to the bare space condition.

**Fig 2:**
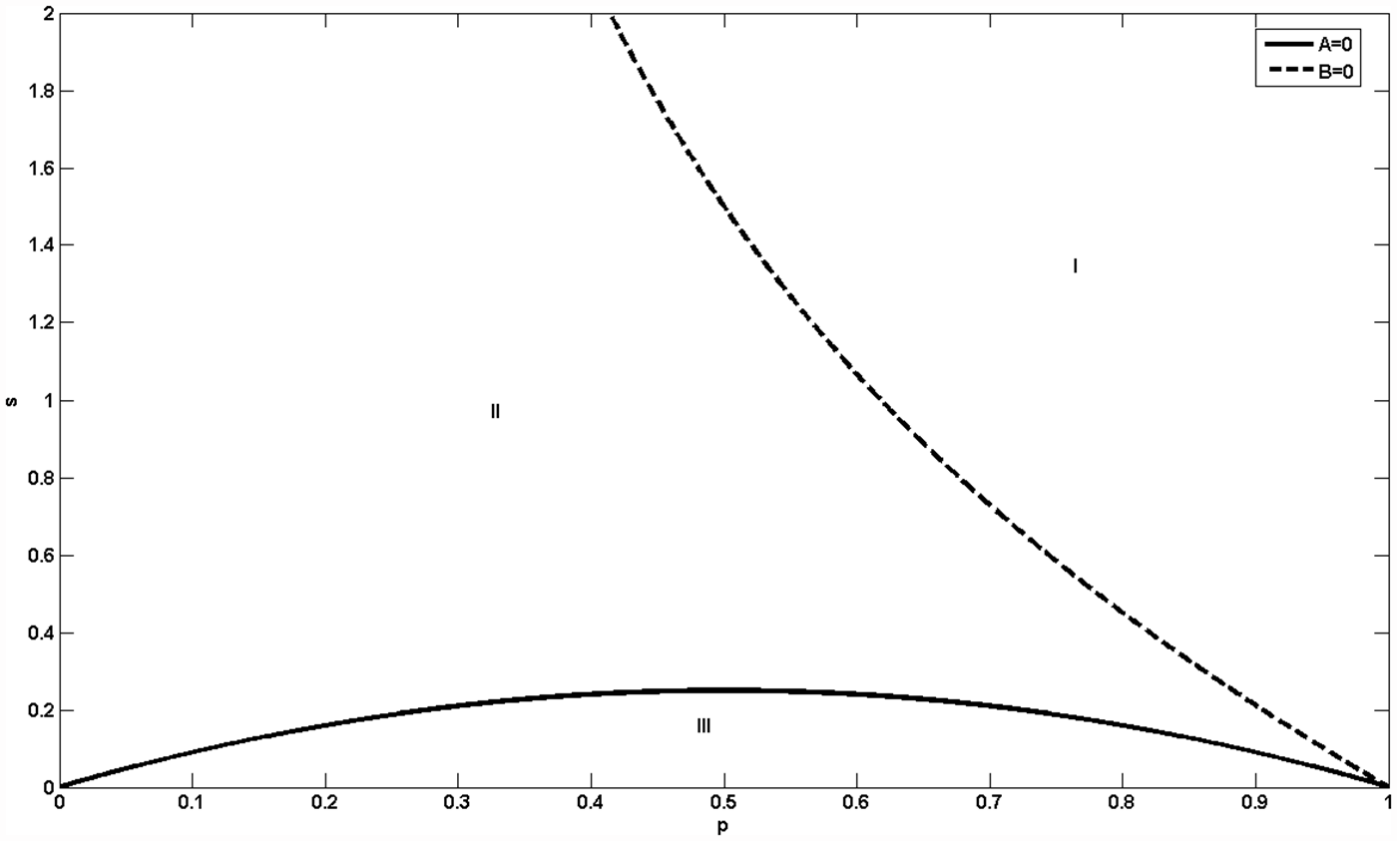
The biodiversity of the Jungle game in the first example. Species *S*_1_, *S*_2_ and *S*_5_ coexist in region I; all five species coexist in region II; and species *S*_1_, *S*_4_ and *S*_5_ coexist in region III.

Let *K*_*i*,*i*+1_ = 1(i = 1,2,3,4) denote the predation rate of a species feeding on its primary food. *K*_5,1_ = *s* denotes the inhibitory rate from the bottom-level species to top-level species and *p*, *p* < 1 denotes the predation rate of a species feeding on its secondary food, and we have *A* = *C* = *p*^2^−*p*+*s* and *B* = *p*^2^+*sp*−1. Fig 2 shows the biological diversity of the jungle game.

In Fig 2, the solid line denotes *A* = *C* = 0 and the dashed line denotes *B* = 0. In region I, species *S*_1_, *S*_2_ and *S*_5_ coexist in the system with *A* > 0 and *B* > 0. In region II, all five species coexist with *AB* < 0 and *AC* > 0. In region III, species *S*_1_, *S*_4_ and *S*_5_ coexist with *B* < 0 and *C* < 0. We can go further to say that if we do not distinguish between the species’ primary food and sub-food (when *p* = 1), the system will degenerate into three species (the two top-level species and the bottom-level species) regardless of the value *s*, as explained in [32]. If species can only feed on their primary food, all five species coexist as the conclusion of the rock-paper-scissors game with more than three strategies. In our work, we investigate the difference between the predation rates of species capturing their primary and secondary food. Because 0 < *p* < 1, the coexistence of species relates to the inhibitory strength from the bottom-level species to the top-level species. If *s* > 1/*p* − *p*, the two top-level species *S*_1_, *S*_2_ and the bottom-level species *S*_5_ coexist. If *s* < −*p*^2^ + *p*, the top-level species *S*_1_ and the two bottom-level species *S*_4_, *S*_5_ coexist. When 1/ *p* − *p < s* < −*p*^2^ + *p*, all five species coexist.

## Example 2

Similar with Example 1, we do not consider the trophic loss in this model. Let *K*_*i*,*i*+1_ = 1(*i* = 1,2,3,4), *K*_*i*,*i*+2_ = *p*_1_ (*i* = 1,2,3), *K*_*i*,*i*+3_ = *p*_2_ (*i* = 1,2), *K*_5,1_ = *s*; we then obtain *A* = *C* = 0⇔*s* = *p*_1_(1 − *p*_2_) and $B=0\Leftrightarrow s=p_{1}^{-1}\left(1-p_{2}^{2}\right)$.

In the parameter space (*p*_2_, *s*), the curve *A* = 0 is a straight line through points (0, *p*_1_) and (1, 0). The curve *B* = 0, through points $\left(0,p_{1}^{-1}\right)$ and (1, 0), is a parabola with its axis of symmetry lying on the y-axis.

**Fig 3:**
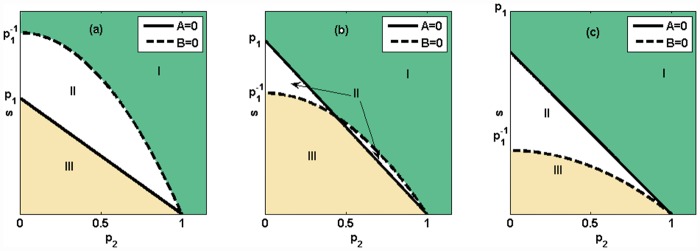
The biodiversity of the Jungle game in the second example. (a) *p*_1_ ≤ 1; (b) $1<p_{1}<\sqrt{2}$; (c) $p_{1}\geq \sqrt{2}$. Species *S*_1_, *S*_2_ and *S*_5_ coexist in region I (green); all five species coexist in region II (white); and species *S*_1_, *S*_4_ and *S*_5_ coexist in region III (yellow).

Fig 3(a) shows the coexistence of the system when *p*_1_ ≤ 1. As we can see from the figure, when *p*_2_ ≥ 1, species *S*_1_, *S*_2_ and *S*_5_ coexist in the system regardless of the value of parameter *s*. When *p*_2_ < 1, the coexistence of the system is related to the value of *s*. A larger or smaller *s* may lead to the extinction of two species. If the value of *s* is appropriate, all five species can coexist, as shown in Fig 3(a).

Fig 3(b) shows the coexistence of the system when $1<p_{1}<\sqrt{2}$. As we can see from the figure when *p*_2_ ≥ 1, species *S*_1_, *S*_2_ and *S*_5_ coexist in the system no matter what the value of parameter *s*. When *p*_2_ < 1 and $p_{2}\neq p_{1}^{2}-1$, the coexistence of the system is related to the value of *s*. A larger or smaller *s* may lead to the extinction of two species. When $p_{2}\neq p_{1}^{2}-1$, if $s>p_{1}\left(2-p_{1}^{2}\right)$, species *S*_1_, *S*_4_ and *S*_5_ coexist in the system.

When $p_{1}\geq \sqrt{2}$, we can see the coexistence of the system from Fig 3(c). As shown in the figure, species *S*_1_, *S*_2_ and *S*_5_ coexist in the system regardless of the value of parameter *s*. When *p*_2_ < 1, the coexistence of the system is related to the value of *s*. A larger or smaller *s* may lead to the extinction of two species. If the value of *s* is appropriate, all five species can coexist, as shown in Fig 3(c).

**Fig 4:**
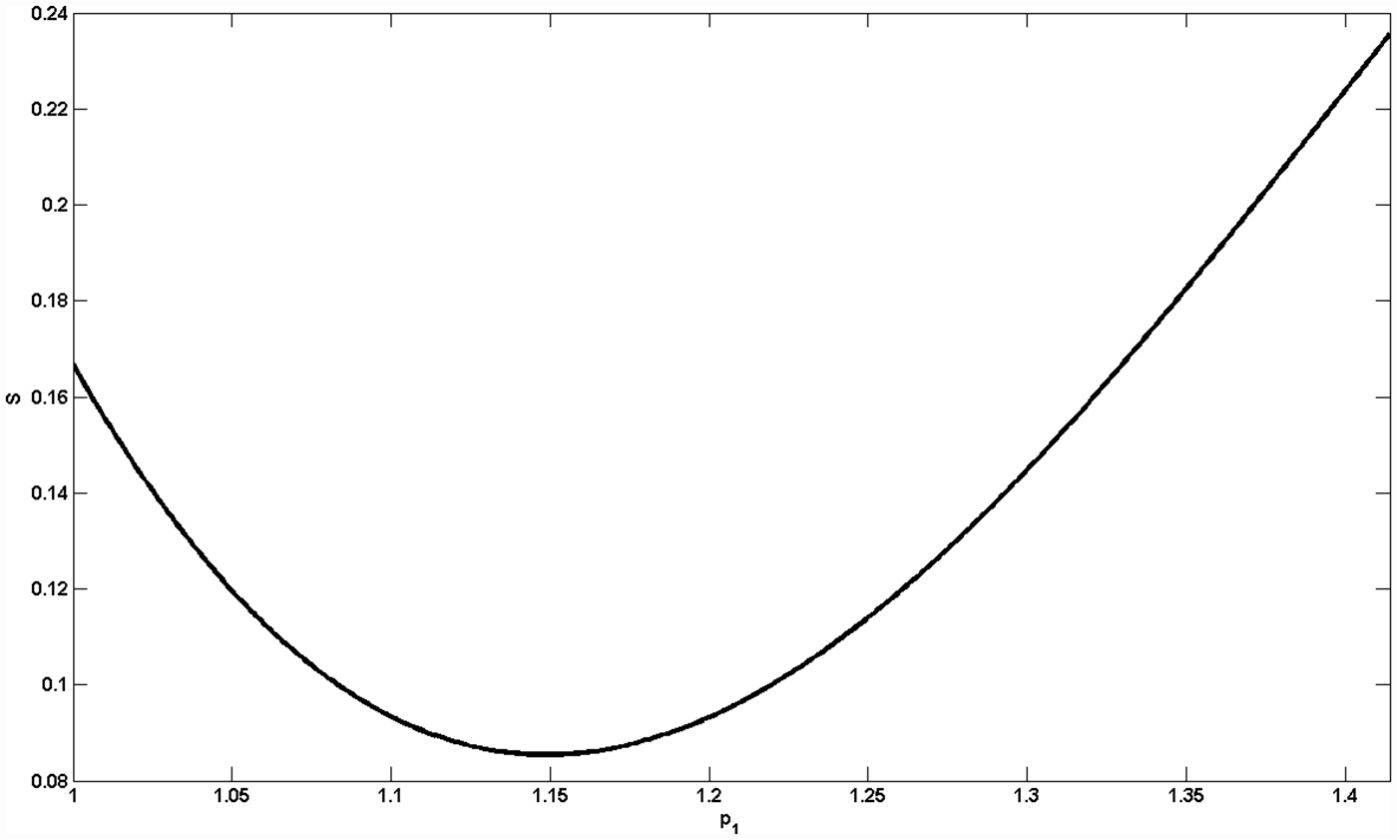
The area of region II in Fig 3 with different *p*_1_. There exists *P* ≈ 1.149 making the smallest area at *p*_1_ = *P*. When *p*_1_ < *P*, the area decreases with the increasing *p*_1_; when *p*_1_ > *P*, the area increases with the increasing *p*_1_.

Let *Area* denote the area of the coexistence region of five species. When *p*_1_ ≤ 1, we get $\frac{\partial Area}{\partial p_{1}}<0$. When $p_{1}\geq \sqrt{2}$, we get $\frac{\partial Area}{\partial p_{1}}>0$. When $1<p_{1}<\sqrt{2}$, the relationship between *Area* and *p*_1_ is shown in Fig 4. In other words, there exists *P* ≈ 1.149 making the smallest *Area* at *p*_1_ = *P*. When $1<p_{1}<\sqrt{2}$, we get $Area=\int_{0}^{1}\left|p_{1}\left(1-p_{2}\right)-p_{1}^{-1}\left(1-p_{2}^{2}\right)\right|dp_{2}=-\frac{1}{3}p_{1}^{5}+2p_{1}^{3}-\frac{7}{2}p_{1}+2p_{1}^{-1}$. As ${\frac{\partial Area}{\partial p_{1}}|}_{p_{1}=P}=0$, we know that *P*^2^ is a root of the equation 10*x*^3^–36*x*^2^+21*x*+10 = 0. Thus, we can go further and say that the closer the predation rate on predators' sub-prey is to 1.149 times the rate on the primary prey, the lower is the area in the parameter space (*p*_2_, *s*) that makes all five species coexist.

## Simulation

Ecological systems exhibit spatial structure; thus, we discuss the Jungle game within the spatial structure. We consider a spatial environment as one that puts individuals on a square lattice of linear size *L* with periodic spatial boundary conditions. Each site can only be occupied by one individual. Interactions between individuals are based on Monte Carlo simulation. Once we randomly place individuals from five species on the lattice, we stochastically choose one individual and one of its Moore neighbors. If the chosen two species are different, letting predator (*S*_*i*_) replace prey (*S*_*j*_) at the probability *k*_*i*, *j*_, we obtain

$$S_{i}+S_{j}\overset{k_{i,j}}{\to }S_{i}+S_{i}$$

where $k_{i,j}=\frac{K_{i,j}}{\sum_{s,t}K_{s,t}}$. The above steps are repeated *L*×*L* times to complete one Monte Carlo step.

**Fig 5:**
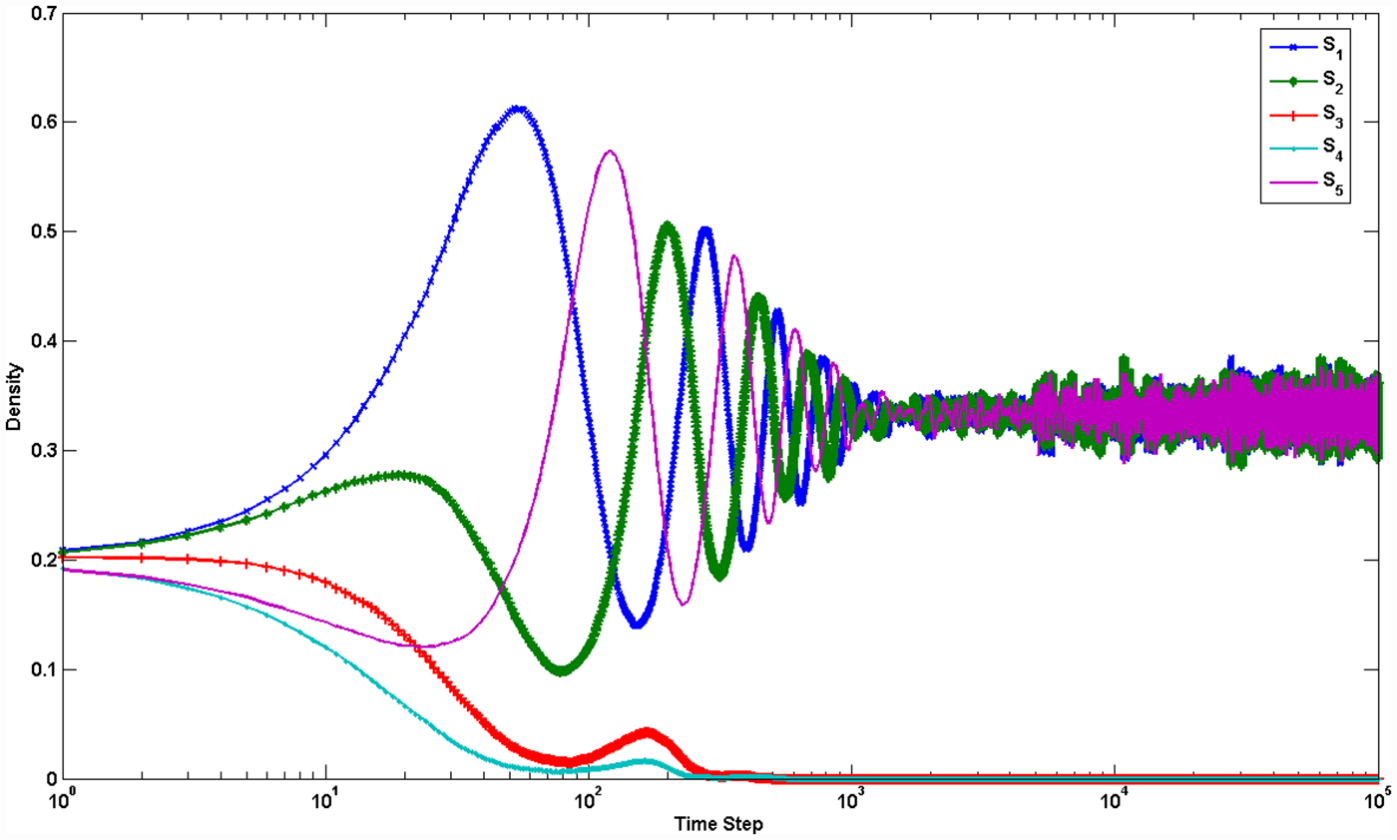
(color online) Densities of five species in Monte Carlo simulation. *L* = 200,*k*_*i*, *j*_ = 1. After about 500 time steps, the species *S*_4_ and *S*_3_ extinct and species *S*_1_, *S*_2_ and *S*_5_ coexist.

Setting *L* = 200 and *k*_*i*, *j*_ = 1, we obtain the steady-state densities of different species evolving through time, as shown in Fig 5. We can see from Fig 5 that species *S*_3_ and *S*_4_ became extinct quickly and that the remaining densities of species *S*_1_, *S*_2_ and *S*_5_ are periodic fluctuations forming a rock-paper-scissors game when setting all values of the invasion rate equal. This result is the same as the results from both Mean-field theory and Ref. [32].

**Fig 6:**
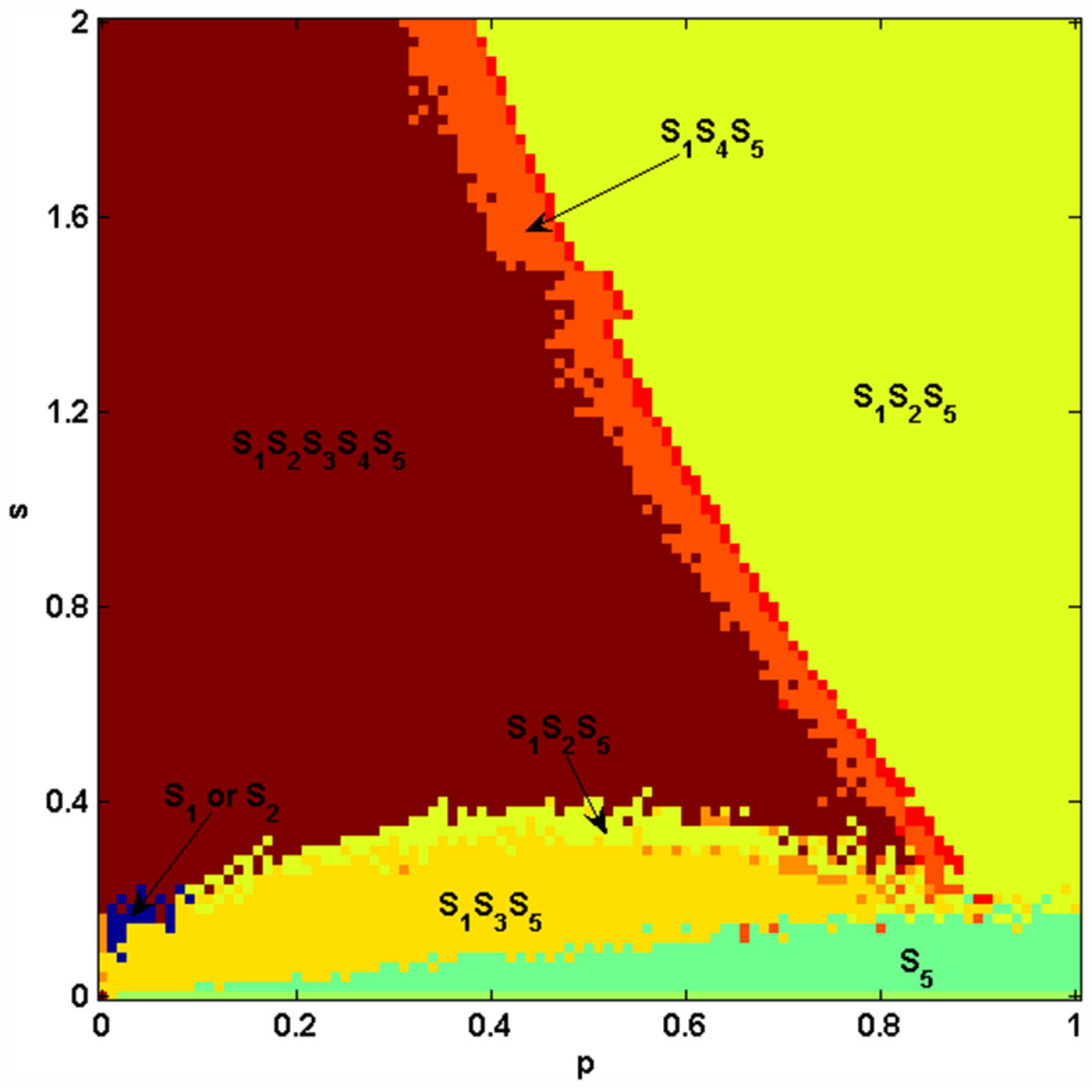
(color online) Coexistence of species in example 1 using Monte Carlo simulation. *L* = 400. All five species coexist in the red region. Species *S*_1_*S*_4_*S*_5_ coexist in the orange region. Species *S*_1_*S*_2_*S*_5_ coexist in the light yellow region. Species *S*_1_*S*_3_*S*_5_ coexist in the deep yellow region. Only *S*_5_ remains in the green region. Only *S*_1_ or *S*_2_ remains in the blue region.

**Fig 7:**
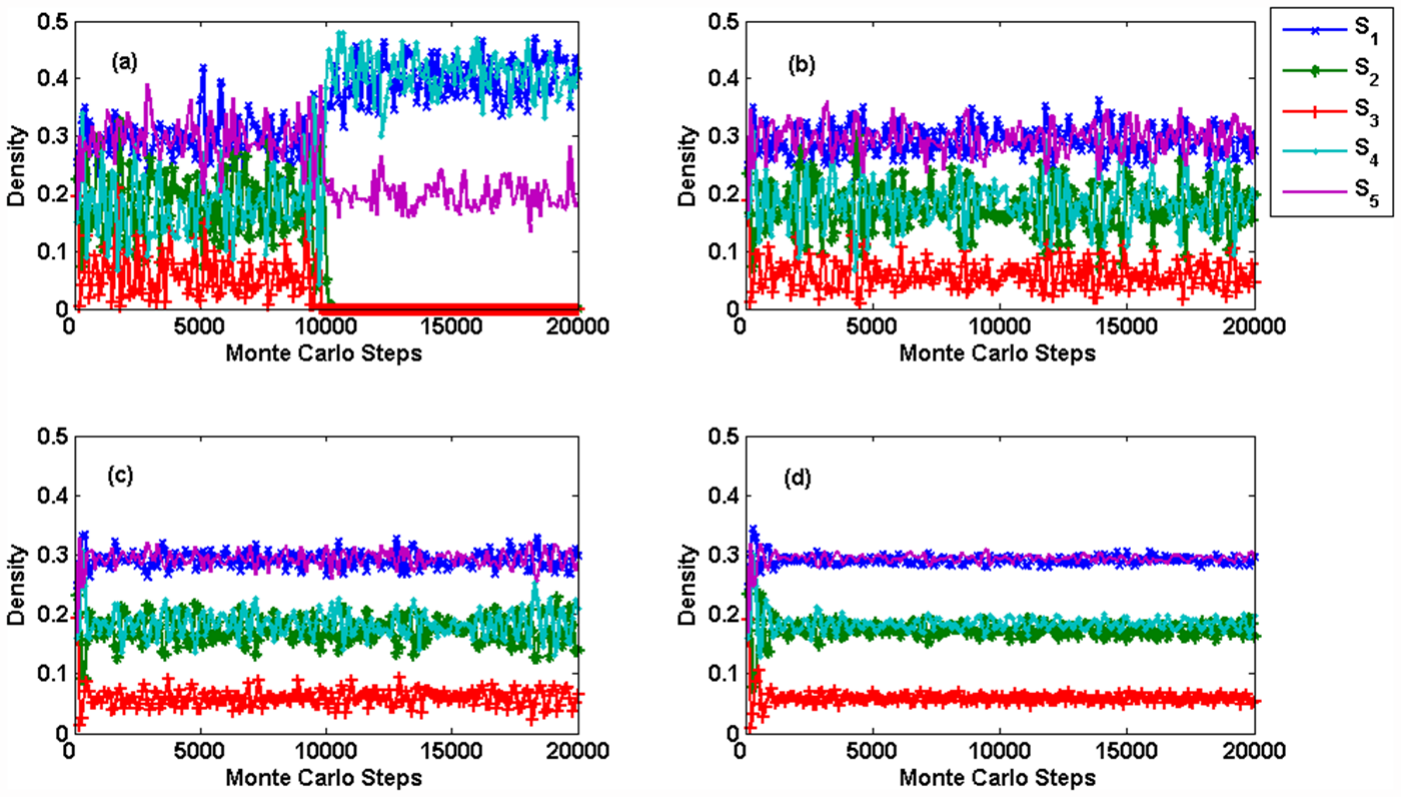
(color online) *p* = 0.5, *s* = 1.2. Densities of species under different population sizes. (a) *L* = 100. (b) *L* = 200. (c) *L* = 400. (d) *L* = 800. (a) Species *S*_2_ and *S*_3_ extinct after 10000 MCS, species *S*_1_, *S*_4_ and *S*_5_ coexist. In (b), (c) and (d), all the five species coexist, the densities fluctuation decreases with the increasing *L*.

**Fig 8:**
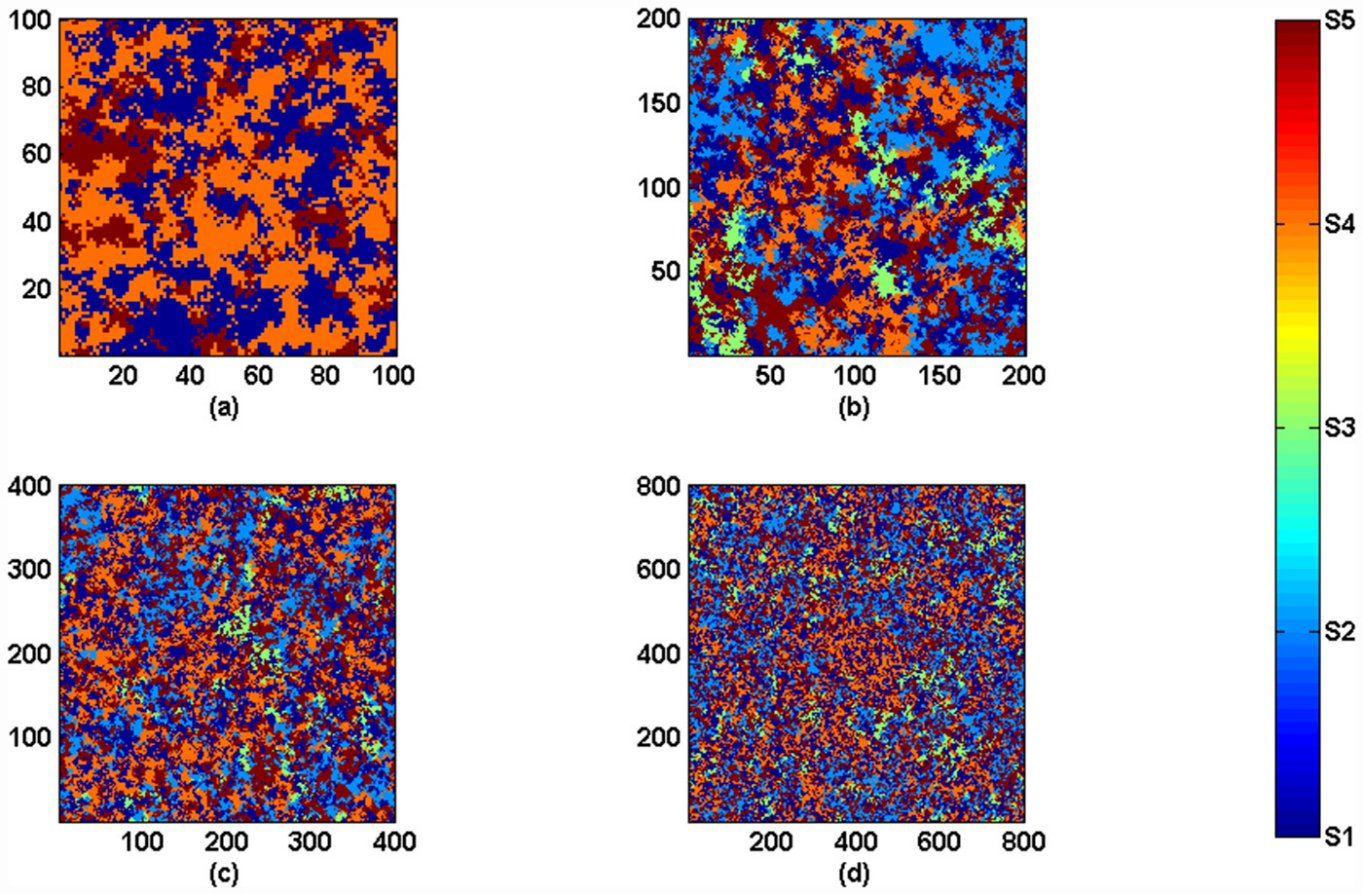
(color online) *p* = 0.5, *s* = 1.2. Spatial patterns after 20000 MCS under different population sizes. (a) *L* = 100. (b) *L* = 200. (c) *L* = 400. (d) *L* = 800. Deep blue represents species *S*_1_, light blue represents species *S*_2_, green represents species *S*_3_, orange represents species *S*_4_ and red represents species *S*_5_.

We traverse the parameters *s* and *p* in example 1. Fig 6 shows the coexistence of species in the Jungle game after 10^5^ Monte Carlo steps under one run. By comparing Figs 3 and 6, we know the coexistence of species in the Monte Carlo simulation, which, considering spatial structure, is different from cases in Mean-field theory, in which species are well mixed. In Mean-field theory, there are only four states of the coexistence of species: all five species coexist, species *S*_1_*S*_2_*S*_5_ coexist, species *S*_1_*S*_3_*S*_5_ coexist and species *S*_1_*S*_4_*S*_5_ coexist. However, in the Monte Carlo simulation with the spatial structure, when the parameter *s* is small, species *S*_5_ may occupy the whole system. Species *S*_1_ or *S*_2_ also may occupy the whole system when both *s* and *p* are small. We also know that the restriction from the bottom-level species to the top-level species has significant influences on the coexistence of species in the ecosystem, both in Mean-field theory and in Monte Carlo simulations. Then, we study the influence of population size on the fluctuation of species’ densities. As shown in Fig 7, the fluctuations of species' densities and the coexistence of species are influenced by different population sizes of species. Species *S*_3_ and *S*_4_ go extinct after 10,000 MCS since *L* = 100, see Fig 7a. When *L* ≥ 200, five species can coexist, as shown in Fig 7b, 7c, and 7d. At this time, the fluctuations of species' densities decrease with the increasing *L*. Huge population size reduces the fluctuation of density. Similar with Ref. [29], the population size has impact on the coexistence of the system. Fig 8 shows the spatial patterns of species under different population sizes *L*. We can see from Figs 7 and 8 that the size of habitat directly influences the biodiversity of the Jungle game. We can go further to say that the loss of habitat may be the major factor leading to the extinction of species.

## Conclusions

We construct a cyclic, five-species competing model with the special topology as the Jungle game. In this model, species are located on different levels. The top-level species can invade all other species except the bottom-level species. The bottom-level species can only invade the top-level species. Other species can invade the levels lower than themselves. We discuss the system stabilities using mean-field theory. The results imply that invasion rates can affect species’ coexistence. We also find that the coexistence of species is related to the invasion rate between species. All five species can coexist under specific invasion rates. This result is different from the results in Ref. [32], which did not consider the impact of a changing invasion rate on the system.

In the first simplified model, our findings have biological significance. In the five-species jungle game, if all of the species only feed on their primary food source, all five species can coexist. If the species have the same invasion rates for their primary and secondary food sources, two species become extinct, and the two top-level species and the bottom species remain and constitute a stable rock-paper-scissors game. If the species prefer to invade the primary food rather than the secondary food, the coexistence of the system relies on the invasion rate by which the bottom-level species inhibits the top-level species. In nature, there can be different ways that the bottom-level species can invade the top-level species. For instance, the forest is destroyed by fire to return to the bare space condition. We find that inhibition from the bottom-level species to the top-level species can be a critical factor that affects biodiversity.

In the second simplified model, we divided the species’ food into three levels. The neighbor-level food is the primary food of a species. Then, the next-level food is the secondary food, followed by the third food. We find that if the predator prefers its third food rather that its primary food, the system of five species cannot coexist. When a predator prefers to hunt its primary prey than its third-level prey, the biodiversity in the Jungle game depends on the invasion rate that the bottom-level species invade the top-level species. Excessive high or low invasion rate will lead to the extinction of species. If a predator prefers to hunt its primary prey than its second-level prey or the preference for a predator to hunt its second-level prey is $\sqrt{2}$ times than that to hunt its primary prey, the decreasing preference for a predator to hunt its third-level prey makes the increasing invasion rate range which made the all species in the system stable coexistence. If a predator prefers to hunt its second-level prey than its primary prey or the preference for a predator to hunt its second-level prey is $\sqrt{2}$ times lower than that to hunt its primary prey, the invasion rate range which made the all species in the system stable coexistence is tiny. The more the predation rate on predators' sub-prey approaches 1.149 times the rate on the primary prey, the lower is the possibility that all five species coexist.

We find more coexistence cases of species in the Monte Carlo simulation, considering the spatial structure: all five species coexist, species *S*_1_*S*_2_*S*_5_ coexist, species *S*_1_*S*_3_*S*_5_ coexist, species *S*_1_*S*_4_*S*_5_ coexist, only *S*_1_ exists, only *S*_2_ exists, or only *S*_5_ exists. The coexistence of species is related to the fluctuation of density and the population size. However, the restriction probability *s* from bottom-level species to top-level species has significant influences on the coexistence of species in the ecosystem, both when considering spatial structure and when not considering spatial structure.

Although we do not attempt to explain the multiple-species ecosystem in the association of species, the cyclically dominant associations are worth further research[29, 42–44]. There are three three-species associations, *S*_1_*S*_2_*S*_5_, *S*_1_*S*_3_*S*_5_, and *S*_1_*S*_4_*S*_5_, and one five-species association, *S*_1_*S*_2_*S*_3_*S*_4_*S*_5_, in our model. Additionally, three species may occupy the entire system by themselves: *S*_1_, *S*_2_ and *S*_5_. Thus, the cyclically dominant association is also an important way to study the Jungle game. Similarly, it should be noted that the models we mentioned in our paper are based on zero-sum games, thus they cannot immediately apply to food-chain models which include trophic loss. Therefore, the Jungle game with the trophic loss is worth to research. In other population dynamics systems, the invasions between species and the transformation relationships are similar to the Jungle game. For example, in some epidemiological models[34, 36], the susceptible ones are similar to the bottom-level species in our paper, the immune ones are similar to the top-level species in our paper. The methods and results we got may be helpful to the researches on these fields.

## Appendix

1. Assume *A* > 0 and *B* > 0. Setting

$$V\left(x_{1},x_{2},x_{3},x_{4},x_{5}\right)\text{=}x_{1}^{K_{2,5}}x_{2}^{K_{1,5}}x_{5}^{K_{1,2}},$$

Thus,

$$\frac{dV}{dt}=V\left(K_{2,5}\frac{1}{x_{1}}\frac{dx_{1}}{dt}+K_{1,5}\frac{1}{x_{2}}\frac{dx_{2}}{dt}+K_{1,2}\frac{1}{x_{5}}\frac{dx_{5}}{dt}\right)=V\left(Ax_{3}+Bx_{4}\right)\geq 0.$$

As *V* ≤ 1 and $\left|\frac{d^{2}V}{dt^{2}}\right|<\infty$, we know that the subspace $\left\{\frac{dV}{dt}=0\right\}$ which means {*x*_3_ = *x*_4_ = 0} is the invariant subspace of (Eq 1). Based on LaSalle’s invariance principle, the subspace {*x*_3_ = *x*_4_ = 0} is also the global attractor of (Eq 1). Hence, species *S*_3_ and *S*_4_ die out.

As *V*(*t*) monotonic is non-decreasing, species *S*_1_, *S*_2_ and *S*_5_ will not become extinct if they exist at the beginning.

2. Assume *A* < 0 and *C* > 0. Setting

$$V_{2}\left(x_{1},x_{2},x_{3},x_{4},x_{5}\right)\text{=}x_{1}^{K_{3,5}}x_{3}^{K_{5,1}}x_{5}^{K_{1,3}},$$

Thus,

$$\frac{dV_{2}}{dt}=V_{2}\left(K_{3,5}\frac{1}{x_{1}}\frac{dx_{1}}{dt}+K_{5,1}\frac{1}{x_{3}}\frac{dx_{3}}{dt}+K_{1,3}\frac{1}{x_{5}}\frac{dx_{5}}{dt}\right)=V_{2}\left(-Ax_{2}+Cx_{4}\right)\geq 0.$$

As *V*_2_ ≤ 1 and $\left|\frac{d^{2}V_{2}}{dt^{2}}\right|<\infty$, we know that the subspace $\left\{\frac{dV_{2}}{dt}=0\right\}$ which means {*x*_2_ = *x*_4_ = 0} is the invariant subspace of (Eq 1). Based on LaSalle’s invariance principle, the subspace {*x*_2_ = *x*_4_ = 0} is also the global attractor of (Eq 1). Hence, species *S*_2_ and *S*_4_ die out.

As *V*_2_(*t*) monotonic is non-decreasing, species *S*_1_, *S*_3_ and *S*_5_ will not become extinct if they exist at the beginning.

3. Assume *B* < 0 and *C* < 0. Setting

$$V_{3}\left(x_{1},x_{2},x_{3},x_{4},x_{5}\right)\text{=}x_{1}^{K_{4,5}}x_{4}^{K_{5,1}}x_{5}^{K_{1,4}},$$

Thus,

$$\frac{dV_{3}}{dt}=V_{3}\left(K_{4,5}\frac{1}{x_{1}}\frac{dx_{1}}{dt}+K_{5,1}\frac{1}{x_{4}}\frac{dx_{4}}{dt}+K_{1,4}\frac{1}{x_{5}}\frac{dx_{5}}{dt}\right)=V_{3}\left(-Bx_{2}-Cx_{3}\right)\geq 0.$$

As *V*_3_≤1 and $\left|\frac{d^{2}V_{3}}{dt^{2}}\right|<\infty$, we know that the subspace $\left\{\frac{dV_{3}}{dt}=0\right\}$ which means {*x*_2_ = *x*_3_ = 0} is the invariant subspace of (Eq 1). Based on LaSalle’s invariance principle, the subspace {*x*_2_ = *x*_3_ = 0} is also the global attractor of (Eq 1). Hence, species *S*_2_ and *S*_3_ die out.

As *V*_3_(*t*) monotonic is non-decreasing, species *S*_1_, *S*_4_ and *S*_5_ will not become extinct if they exist at the beginning.

4. Assume *AB* < 0 and *AC* > 0.

Let *D* = *K*_1,4_*K*_2,3_ + *K*_1,2_*K*_3,4_ − *K*_1,3_*K*_2,4_, *E* = *K*_2,3_*K*_4,5_ + *K*_2,5_*K*_3,4_ − *K*_2,4_*K*_3,5_.

If *A* > 0, we set

$$V_{4}\left(x_{1},x_{2},x_{3},x_{4},x_{5}\right)\text{=}x_{1}^{E}x_{2}^{C}x_{3}^{-B}x_{4}^{A}x_{5}^{D},$$

Thus,

$$\frac{dV_{4}}{dt}=V_{4}\left(E\frac{1}{x_{1}}\frac{dx_{1}}{dt}+C\frac{1}{x_{2}}\frac{dx_{2}}{dt}-B\frac{1}{x_{3}}\frac{dx_{3}}{dt}+A\frac{1}{x_{4}}\frac{dx_{4}}{dt}+D\frac{1}{x_{5}}\frac{dx_{5}}{dt}\right)=0.$$

As −*B* > 0, *C* > 0,

$$D=\frac{K_{1,4}K_{2,4}A+K_{1,2}K_{2,4}C-\left(K_{1,2}K_{3,4}+K_{1,4}K_{2,3}\right)B}{K_{2,4}K_{5,1}-B}>0$$

and

$$E=\frac{K_{2,4}K_{4,5}A+K_{2,4}K_{2,5}C-\left(K_{2,5}K_{3,4}+K_{2,3}K_{4,5}\right)B}{K_{2,4}K_{5,1}-B}>0,$$

we can know that species *S*_1_, *S*_2_, *S*_3_, *S*_4_ and *S*_5_ will not become extinct if they exist at the beginning.

If *A* < 0, we set

$$V_{5}\left(x_{1},x_{2},x_{3},x_{4},x_{5}\right)\text{=}x_{1}^{-E}x_{2}^{-C}x_{3}^{B}x_{4}^{-A}x_{5}^{-D},$$

Thus,

$$\frac{dV_{5}}{dt}=V_{5}\left(-E\frac{1}{x_{1}}\frac{dx_{1}}{dt}-C\frac{1}{x_{2}}\frac{dx_{2}}{dt}+B\frac{1}{x_{3}}\frac{dx_{3}}{dt}-A\frac{1}{x_{4}}\frac{dx_{4}}{dt}-D\frac{1}{x_{5}}\frac{dx_{5}}{dt}\right)=0.$$

As *B* > 0, −*C* > 0,

$$-D=-\frac{K_{3,5}\left(K_{1,4}K_{2,4}A+K_{1,2}K_{2,4}C-\left(K_{1,2}K_{3,4}+K_{1,4}K_{2,3}\right)B\right)}{K_{2,3}K_{4,5}K_{5,1}+K_{2,5}K_{3,4}K_{5,1}-K_{4,5}A-K_{2,5}C}>0$$

and

$$-E=-\frac{K_{3,5}\left(K_{2,4}K_{4,5}A+K_{2,4}K_{2,5}C-\left(K_{2,5}K_{3,4}+K_{2,3}K_{4,5}\right)B\right)}{K_{2,3}K_{4,5}K_{5,1}+K_{2,5}K_{3,4}K_{5,1}-K_{4,5}A-K_{2,5}C}>0,$$

we can know that species *S*_1_, *S*_2_, *S*_3_, *S*_4_ and *S*_5_ will not become extinct if they exist at the beginning.
